# Transcranial brain stimulation (TMS and tDCS) for post-stroke aphasia rehabilitation: Controversies

**DOI:** 10.1590/S1980-57642014DN83000003

**Published:** 2014

**Authors:** Lucia Iracema Zanotto de Mendonça

**Affiliations:** 1 MD, PhD. Mestre e Doutora em Neurologia - Faculdade de Medicina da Universidade de São Paulo, SP, Brazil.

**Keywords:** aphasia, transcranial magnetic stimulation, rehabilitation

## Abstract

Transcranial brain stimulation (TS) techniques have been investigated for use in the rehabilitation of post-stroke aphasia. According to previous reports, functional recovery by the left hemisphere improves recovery from aphasia, when compared with right hemisphere participation. TS has been applied to stimulate the activity of the left hemisphere or to inhibit homotopic areas in the right hemisphere. Various factors can interfere with the brain's response to TS, including the size and location of the lesion, the time elapsed since the causal event, and individual differences in the hemispheric language dominance pattern. The following questions are discussed in the present article:

[a] Is inhibition of the right hemisphere truly beneficial?;
[b] Is the transference of the language network to the left hemisphere truly desirable in all patients?;
[c] Is the use of TS during the post-stroke subacute phase truly appropriate? Different patterns of neuroplasticity must occur in post-stroke aphasia.

## Abstract (pt)

As técnicas de estimulação cerebral transcraniana (ET) têm sido estudadas como recurso na reabilitação da afasia resultante de acidente vascular cerebral. Tem sido apontado que melhor recuperação da afasia ocorre quando o hemisfério esquerdo reassume a função da linguagem, quando comparado à participação do hemisfério direito. A ET pode estimular a atividade do hemisfério esquerdo ou inibir a atividade de áreas homotópicas do hemisfério direito. Vários fatores podem interferir na resposta à ET, como o tamanho e local da lesão, o tempo decorrido do evento causal e diferenças individuais no padrão de dominância hemisférica para a linguagem. Este artigo discute as seguintes questões:

[a] Realmente é benéfico inibir o hemisfério direito?
[b] Realmente é desejável a transferência para a esquerda da função da linguagem em todos os casos?
[c] Realmente é adequada a aplicação da TS na fase subaguda pós AVC? Diferentes padrões de reorganização cerebral devem ocorrer frente à presença de afasia decorrente de AVC.

## INTRODUCTION

Aphasia is a significant sequela of neurological diseases, especially stroke, and recovery differs between patients.

In healthy individuals, language is a complex function that includes the participation of multiple brain areas from both hemispheres. For this reason, language is particularly vulnerable to brain injury. Language exhibits lateralization, and the left hemisphere shows dominance in linguistic skills for 96% of healthy, right-handed individuals.^1^ Nevertheless, human communication is based on a set of phonological, semantic, discursive and pragmatic features, which depend on the integration between left and right hemispheres and interaction with other cognitive functions. These data suggest that the right hemisphere plays a specific role in language. The pattern of hemispheric language dominance is related to laterality^1^ and literacy.^2^ Functional neuroimaging has detected changes in the pattern of brain activation in bi/multilingualism^3,4^ and as a function of age.^5,6^ Thus, the brain circuits associated with language vary based on the life experiences of an individual, and this individualization may influence the reorganization of the neural network that occurs after brain injury.

Two TS methods have been used for the rehabilitation of patients with aphasia, including transcranial direct-current stimulation (tDCS) and transcranial magnetic stimulation (TMS). Both methods are considered safe.^7,8^

Left and right hemispheres can participate in aphasia recovery. Better recovery has been associated with the restoration of function by the left hemisfhere.^9^

**Figure 1:**
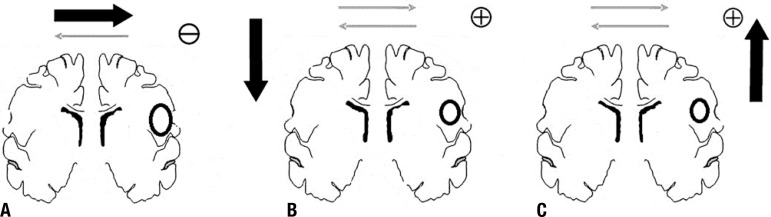
[A] Intact hemisphere may exert high inhibitory influences on the lesioned hemisphere. [B] Inhibitory TS contralateral to the lesion rebalances the interhemispheric interaction. [C] Excitatory TS ipsilateral to the lesion rebalances the interhemispheric interaction.

Models of interhemispheric competition have been described for motor and sensory systems. By extending this concept to the language domain, the intact right hemisphere may exert inhibitory influences on the lesioned left hemisphere and interfere with the reacquisition of efficient language processing through left-hemisphere cortical networks(Figure 1A). There is some support for the hypothesis of reciprocal transcallosal inhibition in language networks.^10^

TS may exert excitatory or inhibitory effects on the underlying brain tissue. Therefore, TS has been used to favor recruitment of left-hemispheric language networks, increasing the activity of the left hemisphere (ipsilateral to the lesion) or disrupting interhemispheric inhibition by downregulating the activity of the right hemisphere (contralateral to the lesion) (Figure 1B and 1C).

However, some authors have argued that the right hemisphere is important for recuperation at least in some patients^9^ and that homotopic areas are not necessarily homologous areas.^11^ Their data suggest that the strategy of promoting functional recovery by the left hemisphere and of inhibiting the right hemisphere may not be effective for all patients.

The purpose of this article is to discuss TS use in the context of the possible mechanisms of network language reorganization. The controversial topics are the inhibition of homotopic areas in the right hemisphere, the use of TS in the subacute phase post-stroke, and methodological aspects of the studies. This article also highlights the need to respect individual differences in the language network prior to the lesion.

## POST-APHASIA FUNCTIONAL REORGANIZATION

After damage to the left hemispheric language network, the functional recovery of aphasia can occur by activation of the perilesional area in the left hemisphere, by recruitment of residual left-hemispheric structures that may have been previously involved in language function, or by activation of the right hemisphere.^12^

The contribution of each brain hemisphere to recovery from aphasia is controversial. The role of the right hemisphere in language recovery and its interaction with damaged left-hemispheric structures has not been elucidated.

## CONTRIBUTION OF THE LEFT HEMISPHERE

Satisfactory recovery from aphasia has been consistently associated with the restoration of left-hemispheric functions. Neurofunctional studies have correlated left lateralization and activation with improved language ability,^13,14^ suggesting that the preservation or restoration of the left hemispheric language network is important for recovery from aphasia.

However, the activation of right-side homotopic areas was also found in individuals who exhibited satisfactory recovery.^11^

## CONTRIBUTION OF THE RIGHT HEMISPHERE

Evidence indicates that the right hemisphere contributes to recovery from aphasia. Children who suffered extensive damage to the left hemisphere or who were subjected to hemispherectomy exhibited significant language recovery.^15^ In adults who displayed satisfactory recovery from aphasia following brain damage to the left hemisphere, subsequent injury of the right hemisphere was able to cause new functional deterioration.^16^ Functional neuroimaging and TMS have shown the transfer of language functions to the right hemisphere in patients with slowly progressing left hemisphere tumors.^17^

The right hemisphere participates in the recovery from aphasia through the activation of homotopic areas, which are analogous in location to the language areas of the left hemisphere.^11^ These areas constitute a useful compensatory network for speech disorders, even if they are computationally less efficient.

However, the activation of these brain areas can be dysfunctional. For instance, right-side homotopic areas may be related to other features of communication and thus result in maladaptive recovery. The contribution of the right hemisphere to recovery from aphasia might be due to its participation in executive function, attention and memory, rather than through direct language restoration.^18^

Some studies have found a relationship between activation of the right hemisphere and aphasia improvement.^9^ Other studies suggest that the right-hemispheric shift as a mechanism of post-stroke recovery in adults is an ineffective way for language function recovery.^19^

Therefore, the functional relevance of the activation of homotopic right-hemispheric language areas remains ambiguous.

## INFLUENCE OF LESION SIZE AND RECOVERY TIMES

Post-injury brain activation patterns depend on the size and extent of the lesion. The recruitment of left perilesional areas with variable involvement of right-hemispheric structures occurs in small lesions in the left hemisphere.^20^ In addition, the participation of the right hemisphere is often significant in large lesions.^21^ However, a significant correlation between lesion volume in the dominant hemisphere and activation of the non-dominant hemisphere has not been determined.^14^ In addition to the activation of left-hemispheric language regions, a robust activation in homotopic right-hemispheric regions regardless of lesion size has been observed.^22^

These data must be assessed as a function of recovery period. Initially, after a stroke, there may be a reallocation of language function to the right hemisphere, particularly in patients with extensive left-hemispheric injury. Over time, this recruitment diminishes and is followed by a redistribution of language processing back to the left hemisphere; however, this process is more likely to occur in patients with relatively small lesions.^9,20^

Changes in the activation pattern of the brain hemispheres over time suggest that the initial temporary increase in right-hemispheric activation does not necessarily reflect a functionally relevant reorganization process. Alternatively, the increased activation may be due to changes in transcallosal inhibition.

Specific participation of the right hemisphere in recovery from aphasia is possible. Activation of the left hemisphere is not reestablished in all cases, especially in individuals with large left-side lesions. Increased activation of the right inferior frontal gyrus (IFG) from the acute to the subacute phase, is associated with improved language performance.^9^ Recruitment of the right hemisphere during recovery from aphasia can be effective if it occurs during a critical time window post-stroke and depends on the lesion's location, extent and permanence.^23^

In summary, the brain mechanisms involved with reorganization during recovery from aphasia are variable and depend on the size and extent of the lesion and on recovery time.

## TS IN APHASIA REHABILITATION

The first studies that used TS in patients with aphasia involved cases of nonfluent aphasia due to stroke. TS techniques have also been used in cases of progressive aphasia.^24^ The following discussion mainly focuses on aphasia secondary to vascular brain injury because degenerative conditions progress slowly and likely result in a different brain reorganization pattern.

TS has been used alone or concomitantly with speech and language therapy; however, both favorable^25^ and ineffective^26^ results have been reported with TS alone. Thus, whether TS should be used alone or combined with additional behavioral treatment strategies remains unclear. TS may further improve aphasia symptoms by potentiating the neural signals elicited by other therapies.

## STIMULATION OF THE LEFT HEMISPHERE

One aim of TS is activation of the left hemisphere.

TS has been applied to Broca's^27-29^ and Wernicke's^29,30^ areas.

TS applied in Broca's area significantly improved naming accuracy,^27^ semantic fluency^28^ and spontaneous speech, as evidenced by the ability to use connective words to establish cohesion among adjacent utterances.^29^

The fMRI maps obtained after TS showed increased activation of the left fronto-temporo-parietal language networks with a significant left-hemispheric shift compared with images taken prior to treatment.^28^

Differences in functional improvement have been found based on the site of TS. A significantly greater improvement in noun naming was found after stimulation of the temporal region, while verb naming significantly improved after stimulation of the frontal region.^30^

## INHIBITION OF THE RIGHT HEMISPHERE

A large number of studies have focused on inhibition of the right hemisphere.

Researchers have particularly focused on the triangular portion of the right inferior frontal gyrus (IFG).

Several aphasia scales show improvements in naming, repetition, reaction time and oral expression and comprehension following the application of right-side inhibitory TS.^25,31,32^ The improvement in naming is particularly significant for action naming.^33^ An improvement in picture descriptions with respect to the number of narrative words and nouns, sentence length, and the use of closed class words has also been described.^33^

However, not all studies have reported favorable results following inhibition of the right hemisphere. Poor outcomes in naming, semantics, fluency and reaction time have also been described,^33-36^ suggesting that the right IFG may play an essential role in the residual language function of some patients.

Naeser et al.^37^ emphasized that the application of inhibitory stimulation to the right *pars opercularis* of the IFG (POp) impaired performance, while the same stimulation of the right *pars triangularis* (PTr) improved performance.^25,37^

Therefore, when TS is applied to the IFG, local anatomical-functional features should be taken into consideration.

Broca's area is located in the posterior IFG (pIFG) and encompasses Brodmann's areas 44 (approximately corresponding to the opercular portion of the pIFG) and 45 (approximately corresponding to the triangular portion of the pIFG). The two regions of right Broca's homolog (the PTr and POp) may be functionally different and might play different roles in aphasia recovery.

Evidence suggests that the right POp plays a causal role in phonologic processing in normal subjects.^38^ Functional imaging studies have shown that the right and left pIFG are activated when healthy right-handed individuals make phonological word decisions^39,40^.

Results from fMRI demonstrate that there is a reliable increase in activation for semantic relative to phonological decisions in the anterior region of IFG (PTr), while the opposite comparison (phonological vs. semantic decisions) shows an area of enhanced activation within the posterior region of IFG (POp).^40^

TMS can be used to temporarily interfere with neural processing in the IFG. TMS applied over the anterior IFG (PTr) significantly slowed subjects' reactions for the semantic tasks,^40^ while TMS of posterior IFG (POp) impaired reaction times and accuracy of phonological decision tasks.^38^ TMS over left, right or bilateral pIFG disrupted phonological processing to a similar degree.^38^

In summary, bilateral POp is related with phonologic processing and left PTr is related with semantic processing.

Wernicke's and Broca's areas are linked by the arcuate fasciculus, which is integrated into the superior longitudinal fasciculus. This dorsal stream is related with phonologic processes.^41^ The uncinate fasciculus connects the anterior and middle temporal lobe and the ventrolateral prefrontal cortex. This ventral pathway is related with the semantic process.^41^

The diffusion tensor imaging (DTI)-based tractography method allows visualization of white matter pathways *in vivo*. Using DTI images, Kaplan et al.^42^ studied the pathways related to subregions of Broca's area – PTr and POp. Almost no fiber tracts were visible between PTr and the dorsal pathway in the left and right hemispheres. In contrast to PTr, 8/8 subjects showed robust fiber tracts between POp and the arcuate fasciculus/superior longitudinal fasciculus in the left hemisphere, and 5/8 participants in the right hemisphere.

Therefore, there are functional differences between the subregions of the IFG. Dorsal stream phonologic processes, in which the POp is involved, may be less lateralized, compared with word-level semantic processes associated with the ventral stream and the PTr. Many, but not all, of these areas are homologous in function.^43^

Turkeltaub^43^ reviewed the literature for fMRI or PET studies employing language tasks in patients with chronic aphasia after stroke and healthy controls, using a validated, quantitative neuroimaging meta-analysis method in order to assess mechanisms of adaptation in aphasia. In aphasic subjects, a bilateral distribution of activation included spared areas of the normal left-hemispheric language network, left-hemispheric areas outside the normal network, and right-hemispheric areas that mirrored the left-hemispheric network in controls.

The greatest likelihood for activation was in the IFG, although bilaterally. In general, the right IFG was more reliably recruited when the left inferior frontal cortex was lesioned, but this effect differed between subregions of the IFG.

The right POp was homotopic and functionally homologous to the control subjects' left POp. The right PTr was homotopic to a left-hemispheric control site but was not functionally homologous. Two different areas of the right PTr were recruited, depending on the lesion location, and the function in both areas was unlike that of the normal left PTr.

Thus, the application of inhibitory stimulation to the right POp impairs performance because it interferes with right POp normal function. Although the studies show that stimulation of the right PTr improves performance,^25,37^ Turkeltaub's results suggest a pattern of adaptation after lesioning in the left-hemispheric language networks that involves variation in the mechanisms of right hemisphere recruitment which depends on lesion location.^36,43^

Therefore, the reorganization of the language network following brain injury varies. Different compensatory mechanisms are allocated depending on which part of the network is disrupted. The activation of the right hemisphere is not necessarily maladaptive. Language recovery after stroke may integrate left- and right-hemispheric brain regions to different degrees during the recovery process. Inhibition of the right hemisphere might hamper language recovery.

Some authors have sought the best point for application of inhibitory TS to the right hemisphere. The stimulation site can be determined by using fMRI during a language task; TS is applied to an area homologous to the site with the greatest activation from the fMRI results^27^. Based on the fMRI results, inhibitory TMS could also be applied to the left hemisphere^44^. An exploratory phase delivered to different sites in the right frontal lobe, preceded and followed by a language task, can select the optimal area for stimulation.^45^

Individualized TS is also controversial. The interference of TS on the possible utilization of a functional architecture by the right hemisphere after left-hemispheric injury is unclear. This fact may be evidenced by Turkeltaub's et al. case.^46^ A woman with chronic non-fluent aphasia showed improved naming after inhibitory TMS of the right hemisphere. fMRI confirmed a local reduction in activity at the TMS target without the expected increase in activity in the corresponding left-hemispheric area. Three months after TMS, the patient suffered a right-hemispheric ischemic stroke that resulted in a worsening of aphasia.

## PATIENT-SPECIFIC DIFFERENCES IN THE LANGUAGE NETWORK

The particular features of the cerebral language network should be taken into consideration when TS techniques are used. For instance, the participation of the right and left hemispheres varies as a function of laterality.

Heiss et al.^47^ used inhibitory TMS in the contralesional IFG together with speech and language therapy in subacute post-stroke aphasia patients. A greater level of recovery in language function on global aphasia test scores was found in TMS-treated right-handed patients compared with sham-treated right-handed patients. Language activation patterns assessed with PET showed a shift of activation to the ipsilesional hemisphere in TMS-treated patients, while sham-treated patients consolidated network activity in the contralesional hemisphere. However, the therapeutic efficiency was doubtful in two cases of left-handed aphasics, although no deterioration of language performance was observed. Both patients exhibited a very small interhemispheric shift.

The individual brain organization language patterns in older adults and illiterate or multilingual individuals have not been taken into consideration in previous TS studies.

## USE OF TS IN SUBACUTE POST-STROKE APHASIA

Most studies have used TS in individuals with chronic aphasia. The findings of these investigations cannot be extrapolated to acute or subacute aphasia because the neural adaptation in aphasia changes over time.

The results from the small number of studies on subacute post-stroke aphasia are contradictory. TS was coupled with speech and language therapy in all of these studies.

Stimulation of the affected left hemisphere during the early post-stroke rehabilitation period did not produce statistically significant differences between patients who received tDCS or sham tDCS with regards to naming accuracy and naming time.^48^

Small group differences in the degree of recovery were found between patients receiving TMS that inhibited the right-hemispheric homolog of Broca's area and control participants.^49^ However, follow-up revealed that severely aphasic rTMS patients demonstrated significantly greater improvement in repetition, compared with patients receiving sham stimulation. This result suggests that inhibitory TS applied to the right frontal language homolog is not effective for all post-stroke aphasia patients, although it may benefit a select group of patients.^49^

Other studies revealed significant clinical improvements in naming, comprehension, token tests and writing using inhibitory TS of the right-hemispheric Broca homolog.^50-52^ Results of positron emission tomography (PET) in these analyses showed increased activation in the left hemisphere after treatment, compared with sham-treated patients, and a shift toward the right hemisphere in the control group. This change in laterality indices may^52^ or may not^50^ be related to clinical improvement. Recovery from aphasia due to the restoration of left-hemispheric functioning as shown by a shift in the activation pattern toward the left on PET would be a promising sign. However, this type of transference is not necessarily correlated with clinical improvement and therefore the true significance of this process during the subacute stage of brain injury is not clear.

Winhuisen et al.^53,54^ studied the extent to which the activation of the right IFG is essential for language performance in subacute poststroke aphasia, at different times. They used TMS stimulation to interfere with the function of right and left IFG on a semantic task. At 2 weeks after left hemispheric stroke,^53^ PET activations of the IFG were observed to the left (3 patients) and bilaterally (8 patients). Right IFG stimulation increased reaction time latency or error rate on the semantic task in 5 patients, indicating that in some poststroke aphasics, right IFG activation is essential for residual language function. To test whether the right IFG remained essential for language performance, they reexamined 9 patients, 8 weeks after stroke.^54^ Language function had improved in all patients. At this timepoint, PET activations of the IFG were observed to the left (2 patients) and bilaterally (7 patients). TMS over the left IFG interfered with the language performance in all patients, indicating that the left IFG remained essential. Stimulation over the right IFG interfered with the language performance in 2 patients. Two patients with positive TMS effects over the right side in the initial study did not show these effects at follow-up. The authors suggest that restoration of the left hemisphere network seems to be more effective for aphasia recuperation.

Research on the neuroplasticity following brain injury in animals has shown that commencing training soon after injury^55-58^ and a high degree of stimulation^56,59,60^ hindered recovery by exaggerating excitotoxicity in the vulnerable perilesional tissue. These data suggest the presence of time-and intensity-dependent brain vulnerability.

Caution is required when extrapolating experimental animal data to clinical conditions. The majority of current neuroplasticity knowledge concerns the primary motor, sensory, auditory and visual cortices, which are functionally quite different from language. The ideal time to start a therapeutic intervention in rats was 14 days post-lesion.^59^ Rats typically have a lifespan of 2-3 years; thus, 14 days post-lesion may be equivalent to a longer therapeutic window in humans. In humans, reorganization of the brain circuits and clinical recovery occur spontaneously in 2-3 months.^61^

Evidence from basic science contradicts the general and accepted clinical evidence. The aphasia treatment literature shows that therapy should be started as soon as possible^62^ and that intensive treatment over short periods of time is better than less-intensive regimens over a longer period of time.^63^

The early onset of rehabilitation therapy might prevent disuse and dysfunctional plasticity.

Basic science has shown that neural circuits that are not used for some time become inactive and suffer degradation.^64^ Thus, if disuse hinders recovery, then therapy might preserve the cortical function representation.

The interaction between the brain's adaptation to damage (spontaneous recovery) and therapy over time is another factor that should be taken into consideration. A brain that one may attempt to reorganize with rehabilitative training is one that is being, and likely already has been, driven to reorganize by compensatory behavioral changes.^65^ In this regard, early commencement of rehabilitation therapy should improve recovery.

In an attempt to combine clinical and experimental data, previous work began rehabilitation therapy at a lower intensity which was then increased gradually over time.^58^

The aforementioned treatment considerations account for the introduction of speech and language therapy. The following issues should be considered when using TS for acute or subacute aphasia:

- The method may produce overstimulation at a critical stage of brain injury evolution. The neural signal sent by speech and language therapy is likely less intense than the signal produced by TS, although may help to maintain the cortical function representation.
- Otherwise, TS may force the left hemisphere to reassume the language function, which appears to produce better results. Enhanced activation of the right hemisphere can be observed within 2 weeks after stroke and may return to control levels after 1 year, whereas left-hemispheric activity increases gradually over months to years.^9^ These data suggest that the early application of TS is beneficial. However, the functional meaning of the changes in activation between brain hemispheres remains unknown. Previous studies^9,21,43,53^ suggest that right-hemispheric regions may beneficially contribute to the recovery of a subset of patients; thus, it is important to identify which patients should receive early TS.

Further investigation of TS as a treatment during the acute or subacute phases of post-stroke aphasia is needed.

## METHODOLOGICAL ASPECTS

Methodological questions should be mentioned.

Control groups are lacking in some studies.^25,28,29,33,44^ The participant's performance is analyzed before and after TS application series. The placebo effect could explain the improvement observed.

Language performance is not assessed using a formal test battery in some studies. Naming,^25,32,48,66^ word repetition,^67^ semantic decision,^19^ spontaneous elicited speech^29^ are considered outcome measures after TS.

The authors do not report change of test material, e.g. for picture naming, before and after TS. Consequently, a learning effect could be possible.

Some studies highlight the need for more thorough research.

Elsner et al.^8^ assessed the effects of tDCS with respect to improving aphasia in patients after stroke. These authors included only randomized controlled trials and randomized controlled cross-over trials. No studies used a formal outcome measure for measuring functional communication, and correct picture naming was used as a surrogate for aphasia. There was no evidence that tDCS enhanced speech and language therapy outcomes. The authors concluded that there is no evidence for the effectiveness of tDCS (anodal tDCS, cathodal tDCS) versus control (sham tDCS) in post-stroke aphasia. However, it appears that cathodal tDCS of the non-lesioned hemisphere may be the most promising approach.

Wong and Tsang^7^ performed an evidence-based review of the literature as to the effectiveness of TMS on post-stroke aphasia. The controlled trials showed a positive effect of TMS, with or without conventional rehabilitation, on post-stroke aphasia when compared with sham or conventional rehabilitation alone. However, the authors emphasized that concerns over the methodology of the selected studies warrant a larger-scale, multicenter, well-designed randomized controlled trial involving different phases and types of aphasia before recommending rTMS as a complementary treatment for post-stroke aphasia.

## CONCLUSION

The clinical use of TS methods in post-stroke aphasia rehabilitation remains controversial.

Before the clinical use of TS can be recommended, the following pertinent questions must be answered:

- **Is inhibition of the right hemisphere truly beneficial?** Behavioral evidence confirms that compensatory reorganization occurs within the right hemisphere after the original stroke.
  Homotopic brain areas are not necessarily homologous in their function. The involvement of certain right-hemispheric areas may support recovery in a subset of patients,^46^ especially in cases of large left-side lesions; however, there may not be a correlation between lesion volume in the dominant hemisphere and activation in the non-dominant hemispheric counterparts.^14,22^
- **Is the transference of the language network to the left side truly desirable in all cases?** The brain adaptation that occurs in post-stroke aphasia constitutes a dynamic and progressive process. Increased activation of the left hemisphere after treatment, as quantified by PET, may not correlate with clinical improvement.^31^ The functional meaning of the changes in activation between the brain hemispheres at different stages of clinical evolution in patients is unknown. The meaning of right-hemispheric activation in patients with small lesions that retain some original level of activation is not clear.
- **Is the application of TS during the post-stroke subacute phase truly appropriate?** Some studies on neuroplasticity point to the presence of time- and intensity-dependent brain vulnerability. Applying TS too soon after the occurrence of brain injury and with a high degree of stimulation have been shown to hinder recovery.
  The application of TS in the post-stroke subacute phase can produce overstimulation and excitotoxicity, both of which are detrimental for recovery. There are no reports in the literature that compare the long-term progression of individuals subjected to TS in the subacute and chronic phases.
- **Can TS protocols not consider the variables age, gender, laterality, literacy and bi/multilinguism?** These variables are linked to particular language network and possibly different mechanisms of reorganization after lesion. Patient-specific factors result in differential recruitment from individual to individual.^43^
  Aphasia recovery is associated with a complex pattern of brain reorganization,^68^ involving both ipsilateral and contralateral brain regions, modulated by lesion size and site, time post-onset, training type, and language task.^69^ The differences in recovery mechanisms may be dependent on which part of the network is disrupted.^43^ Factors such as language aspect affected, the degree of language lateralization, age, gender and literacy should be included in TS protocols. The use of TS for recovery from post-stroke aphasia is highly promising; however, future studies with larger patient groups are needed before recommending this method for clinical use.
